# Omic Technologies in HIV: Searching Transcriptional Signatures Involved in Long-Term Non-Progressor and HIV Controller Phenotypes

**DOI:** 10.3389/fimmu.2022.926499

**Published:** 2022-07-01

**Authors:** Erick De La Torre-Tarazona, Rubén Ayala-Suárez, Francisco Díez-Fuertes, José Alcamí

**Affiliations:** ^1^Acquired Immunodeficiency Syndrome (AIDS) Immunopathology Unit, National Center for Microbiology, Institute of Health Carlos III, Majadahonda, Spain; ^2^Centro de Investigación Biomédica en Red de Enfermedades Infecciosas, Instituto de Salud Carlos III (ISCIII), Madrid, Spain; ^3^Departamento de Biomedicina y Biotecnología, Universidad de Alcalá, Alcalá de Henares, Spain; ^4^Human Immunodeficiency Virus (HIV) Unit, Hospital Clínic de Barcelona, Barcelona, Spain

**Keywords:** transcriptome (RNA-seq), HIV infection, long-term non-progressor (LTNP), HIV controllers (HIC), omics, immune cells

## Abstract

This article reviews the main discoveries achieved by transcriptomic approaches on HIV controller (HIC) and long-term non-progressor (LTNP) individuals, who are able to suppress HIV replication and maintain high CD4+ T cell levels, respectively, in the absence of antiretroviral therapy. Different studies using high throughput techniques have elucidated multifactorial causes implied in natural control of HIV infection. Genes related to IFN response, calcium metabolism, ribosome biogenesis, among others, are commonly differentially expressed in LTNP/HIC individuals. Additionally, pathways related with activation, survival, proliferation, apoptosis and inflammation, can be deregulated in these individuals. Likewise, recent transcriptomic studies include high-throughput sequencing in specific immune cell subpopulations, finding additional gene expression patterns associated to viral control and/or non-progression in immune cell subsets. Herein, we provide an overview of the main differentially expressed genes and biological routes commonly observed on immune cells involved in HIV infection from HIC and LTNP individuals, analyzing also different technical aspects that could affect the data analysis and the future perspectives and gaps to be addressed in this field.

## 1 Introduction

Human immunodeficiency virus type 1 (HIV-1) infection is still a major problem of global public health, due to the elevated number of new infections and deaths caused every year, in spite of the tremendous efforts in research and implementation of preventive and therapeutic approaches. Despite that antiretroviral therapy (ART) can control viral infection effectively, its administration is not able to cure the disease. From its discovery to the present, HIV has become one of the most widely studied infectious agents. However, the HIV research field still faces some major challenges such as the achievement of a preventive vaccine or a functional/sterilizing cure.

Several strategies have been proposed to achieve a functional cure for HIV infection. One of them consists of the characterization of factors involved in the control of infection that occurs naturally in a small group of HIV-positive individuals. Immunotherapies able to mimic their patterns of immune response could eventually lead to a functional cure (1). Long-term non-progressor (LTNP) and HIV controller (HIC) individuals can control HIV infection for several years, maintaining high levels of CD4+ T lymphocytes and controlling viral replication, respectively, in the absence of ART and without developing AIDS-related events. However, LTNP/HICs patients represent a very heterogeneous group of patients which are defined by different criteria: viral load (VL), CD4+ T cell counts, and follow-up time (2).

LTNPs comprise between 1-5% of all HIV-1 infected patients. Definition criteria for these individuals are to remain asymptomatic, present CD4+ T cell counts in peripheral blood higher than 500 cells/μL and usually VL in blood below 10,000 copies of viral RNA/ml (3). Follow-up time has been established between 7 to 10 years from the detection of the infection (4). A different group corresponds to viremic non-progressors, a term that is used in the literature in those exceptional patients with the ability to maintain CD4+ T cells over 350 per µL, VL above 10,000 cp/ml, and no ART during follow-up (5), similarly to SIV infection observed in sooty mangabeys (6).

HICs can maintain low or undetectable VL during prolonged periods without ART, and they are classified as elite controllers (EC) (undetectable VL), and viremic controllers (VC) (50-2,000 copies of viral RNA/mL of plasma) (7, 8). The estimated prevalence of EC ranges from 0.15% to 1% among people living with HIV (9–11). HICs generally maintain CD4+ T cell counts over 500 cells/mm^3^, however those that overflow this threshold show a progressive decrease in CD4 numbers (12), perhaps as a consequence of immunological activation and early cellular senescence derived from infection (13).

Understanding the mechanisms involved in these phenomena may be essential to achieve long-term viral remission or new HIV cure strategies. Factors able to reduce the severity of the primary stages of infection in LTNPs and HICs include viral (strains of reduced virulence/fitness) and/or host (innate and adaptive immunity) factors. Regarding viral factors, highly attenuated viral strains with mutations in nef, vpr, and env genes, have been reported (14–16). Additional defective viral factors such as decreased efficiency for viral entry (17), reduced viral fitness (18), defective proviral sequences (19), and transcriptionally inactive provirus integrated into genetic deserts (20), have been described in HIV strains collected from these patients. On the other hand, host factors include genetic variants or differential expression of restriction factors, as APOBEC3 (21), SLFN11 (22), TRIM5A (23), CDKN1A/P21 (24, 25) cytokines, as MIP1A/B (26) and SDF1A (27); and cytokine receptors, as CCR5 (28–30), CCR2 (30), CXCR6 (31) and CX3CR1 (32). In addition, the human leukocyte antigen (HLA) region is key to modulate the presentation of HIV peptides on the surface of infected cells. HLA-I molecules are recognized by CD8+ T and natural killer (NK) lymphocytes, to trigger cytotoxic responses. The HLA alleles -A*25, -B*14/C*0802, -B*2705, -B*5201, -B*5701, -B*5703, -B*5801, -B*8101, B*39 (29, 33, 34), among others, have been associated with the control of infection in LTNP and/or HIC individuals. Finally, immune cell functionalities (proliferative capacity, polyfunctionality, and/or cytotoxic responses) are improved in LTNP/HIC individuals (35, 36).

The systems biology approach studies relationships between individual components and synergistic effects of their interaction in specific biological conditions and it is an innovative strategy to analyze and discover new factors involved in the control of the virus replication in the host. For this purpose, “omics’’ technologies allow the study of a large number of molecules involved in the functioning of organisms. Among “omics” technologies, genomic, transcriptomic, and to a lesser extent, proteomic studies have been applied to the study of extreme phenotypes of HIV-positive individuals. Transcriptomic studies in immune cells from HIV-infected individuals provide the transcriptional landscape in response to the infection of target cells (37, 38). Earlier transcriptome analyses have described that HIV infection causes changes in the expression of genes involved in cell division, transcription, translation, splicing, and cholesterol biosynthesis. These studies have also identified cellular genes involved in permissiveness and resistance to infection, viral replication (39–41), and CD4+ T cell decay (42). The search of differences between typical HIV progressors (TP) and LTNP/HIC individuals could provide relevant knowledge to identify new targets and develop new therapies aimed at a functional cure.

In this review, the main findings from transcriptomic studies performed in immune cells from LTNP and HIC individuals (**Supplementary Tables 1**, **2**) will be discussed in more detail. This work does not pursue being a systematic and exhaustive review of the transcriptomic studies of these patients. We aim to highlight the most representative results which provide important clues about gene expression and biological routes involved in spontaneous viral control/non-progression during HIV infection and to underline future applications of RNA-Seq technology.

## 2 Transcriptomic Studies

One of the most crucial technical aspect to describe a transcriptional signature in a determined phenotypical state is the selection of the target cell population. The transcriptome of peripheral blood mononuclear cells (PBMC) from HIC/LTNPs is well characterized, as they are a good immune model and easier to obtain compared to other cell populations. Research work in PBMC shows the bigger picture of what is happening at distinct cell compartments, which is useful at the first stage for characterizing a gene expression pattern associated with viral control. All the same, it is important to note that cell populations are not equally distributed, and some cell populations with greater representation in the sample may mask some events associated with minority populations. In addition, these transcriptomic signatures cannot be ascribed to a particular cell population, and cell-specific signatures could not be registered on account of reciprocal shifts. Likewise, CD4+ and CD8+ T cells’ transcriptomes have been extensively studied because they are the main target of HIV and cell compartment that displays cytotoxic activity on HIV-infected cells, respectively. However, the investigation of the transcriptome in other immune cells populations, as B cells, NK cells, monocytes, or DCs, has not been performed profusely. For this reason, we have divided the present review into sections, related to the target population of each work. Hence, the goal of this approach is to associate the molecular mechanisms responsible for HIV restriction with their corresponding cell population. Additionally we have added two more sections covering the data integration from different cell populations and the similarities among them.

### 2.1 Whole Blood and Peripheral Blood Mononuclear Cells

Typically, the first attempt to describe mechanisms in AIDS immunopathogenesis relies on using PBMC. These samples are easy to obtain, process, and store with minimal repercussions on patients’ health. In addition, PBMCs are composed of monocytes and lymphocytes, which actively participate in HIV replicative cycle and AIDS immunopathogenesis (43). For this reason, studying PBMCs represents the first natural step to discern the mechanisms of control in these patients, and in this section, we review some case studies using PBMCs, in addition to some studies using whole blood samples.

One of the most overrepresented features found in HIC/LTNP individuals is a different regulation of interferon (IFN) response and by consequence the modulation of the expression of interferon-stimulated genes (ISG). Comparison of PBMC transcriptomes from LTNPs versus TPs, showed a higher expression of nine ISGs (*CMPK2, IFI27, IFI6, IFITM3, NIPAL2, SERPING1, SIGLEC1, DCP1A, NDRG4*) that were associated to non-progression independently of the VL of LTNPs (44). In agreement with these data, gene expression analysis of PBMCs from HIC and ART-treated patients stimulated with HIV peptides *in vitro* displayed a significant upregulation of genes related to the IFN-pathway (*IFIT1, IFI44L, IFI44, MX1, OAS3*) (45). The recurrent appearance of IFN genes and ISGs highlights its importance in AIDS immunopathogenesis, as the generic immune response to viral infection with *OAS* and *MX* genes, or genes specifically involved in HIV infection as *IFI27*. In contrast, in viremic non-progressors lower expression of ISG is found when compared to TPs (46) pointing to a mechanism of viral tolerance instead of the increased and efficient IFN response observed in LTNPs and HIC patients.

Regarding inflammatory responses, most of the scientific literature concur in low-level inflammation in HICs (45, 47) and LTNPs (46, 48). Analyzing whole blood transcription levels, there was a general down-modulation of genes related to inflammation response, like *TLR1, TLR4, TLR6, TLR8, CD14, TREM1, TYROBP*, or *IL-8/CXCL8*) in HICs versus ART (TP-ART) (45), and a decrease in the expression of *IL6* in viremic non-progressors when compared to TPs (46). In PBMCs from ECs, repression of tumor necrosis factor-related apoptosis-inducing ligand (TRAIL) and its short variant, which plays as an antagonist of TRAIL, was described as a consequence of lower toll-like receptor (TLR) activation, leading also to reduced inflammation levels (47). Remarkably, when PBMCs obtained from HICs and TP-ARTs were stimulated with HIV peptides, HICs downregulate several genes related to inflammatory immune response (*CD14, TREM1, IL6, CXCL5, IL1B, IL1A*) at similar levels as unstimulated cells condition (45).

Cell cycling, survival, and apoptosis appear also as central functions in protection against HIV. Molecular cascades involved in these processes are closely related to the HIV replication cycle, thus the modification of their components may be detrimental on viral success. The pathway enrichment analysis based on differentially expressed genes (DEG) of resting PBMCs from LTNP revealed a signature of 11 upregulated genes controlling T cell differentiation, survival, and proliferation (as in *HSH2D* or *STAT5B*), and downregulation of immune cell trafficking, and inflammatory response (48). The most interesting feature of these LTNPs is a positive balance in cell life, upregulating gene expression related to cell survival or anti-apoptotic pathways (*HIG2, GSK3B, TCF4* in Wnt/β-catenin pathway), and hindering cell death or pro-apoptotic pathways *via FOXO3A* downregulation. Another functional enrichment analysis performed on DEG between EC and non-treated TP (NT-TP) showed strong deregulation in cell surface receptor signaling pathways, including molecular cascades of cell death, response to cytokines, and cytokine signaling, in EC individuals (49). Finally, lower expression of apoptosis activator *TRAIL* in ECs (47) would produce a misbalance in apoptosis homeostasis, inducing a non-productive infection state.

Attending to host proteins with major roles in the HIV cycle, *SIGLEC-1* and *CXCR6* genes were downregulated in PBMC from EC when compared to NT-TP (49). *SIGLEC1* is mainly expressed by macrophages and dendritic cells (DC) (50) and plays a role in cell-to-cell HIV transmission, while *CXCR6* can act as a minor co-receptor for HIV-1 entry into the cell (31). The downregulation of these proteins could play a role in the control of reservoir size.

Other PBMC analyses performed by several groups have assessed the microRNA (miRNA) level of expression in HIV natural resistance. Reynoso et al. studied the expression profile of miRNA in plasma samples (51). Attending to the differences between EC and TP-ART, they found an upregulation of miR-29b-3p, miR-146a-5p, and miR-33a-5p in EC patients, and their overexpression in MT2 cells reduced p24 production. Direct targeting of p24 by miR-29b-3p can explain the reduction in protein level and miR-33a-5p might be inhibiting the translation of JNK, burdening the phosphorylation of HIV integrase and hindering the integration of HIV-1 provirus (51). Another study correlated the overexpression of miR-29a, miR-150, miR-31, and miR-31-5p levels with CD4+ T cell counts in EC (52). In addition, miR-125b and miR-150 were downmodulated in EC compared to viremic patients. Concurrently, activated PBMCs from ECs presented the upregulation of miR-29b, allegedly targeting HIV-Nef and HIV-LTR (53). These findings suggest a prominent role of miR-29 family members in HIV natural control of the infection. Our group first assessed the miRNome of PBMC from LTNP through miRNA-Seq (54). We identified 14 DE miRNAs between the LTNP group (including EC and viremic individuals) and typical progressors, with downregulation of miR-144-3p, miR-144-5p, miR-18a-5p, and the upregulation of miR-99b-5p. The subsequent target query and signaling pathway enrichment analysis revealed that phagosome, antigen processing/presenting routes, antiviral response, and intracellular transport contained a significant number of genes regulated by those DE miRNAs.

### 2.2 T Cell Populations

Regarding T cells, the research works usually focus on CD4+ or CD8+ specific populations. Noteworthy, a study conducted by Salgado et al., explored the transcriptome of CD3+ T cells from LTNP and ART-naïve TP (55). They found 136 and 322 upregulated in LTNPs and TP, respectively. Gene ontology categories enrichment analysis with upregulated genes in LTNP revealed cell surface receptors linked to signal transduction (*PDPK1, TLE4, SHOC2, IL17RA, IL1RAP, BMPR2*), and actin cytoskeleton reorganization and biogenesis (*PDPK1, ROCK1, RDX, PDGFB, KLHL5*), as relevant biological processes. DEG-enriched KEGG pathways also point towards the same processes as cytokine-cytokine receptor interaction, regulation of actin cytoskeleton, focal adhesion processes, or negative regulation of apoptosis. This transcriptomic signature of LTNP renders a plausible infection-resistant expression profile, enhancing indirect inhibitors of actin polymerization and thus impairing viral trafficking within the cell. Also, the upregulation of cytokine receptors and adhesion molecules may regulate the activation of T cells and thus be related to the improvement of HIV-specific T cell response. All these biological processes are of relevance in CD4+ and CD8+ T cells, as they will appear in the following sections.

#### 2.2.1 CD4+ T Cells

Numerous studies have focused on CD4+ T cells, as they are the main cell type target for HIV replication. As they drive the immune response, most DEG found in CD4+ T cells intervene in immune response activation. For instance, the upregulation in LTNP of MAPK p38 pathway (*MEK3*), NFKB activation (*NFKBIA*), JNK/MAPK (*JNK2, MKK7*), and granule cell survival pathways altogether with overrepresentation of DEGs in AKT and WNT molecular cascades, contribute to stronger antiviral responses with Th1 differentiation, IFN-γ production and cytotoxic activity (56). Gag-reactive CD4+ T cells from EC and VC patients showed the upregulation of leukocyte migration and positive regulation of CD4+ T cell activation in comparison with chronic progressors (57). Additionally, in this study, EC patients showed a marked Th1 and Th17 response, while viremic patients (VC and TP) presented a predominant T follicular helper (Tfh) and B cells-driven response. Regarding this fact, the CD4+ T cells from HIC patients with a production of broadly neutralizing antibodies were strongly enriched for genes involved in Tfh cells and Th1 responses (58). Altogether, these studies outline the relevance of CD4+ Th1 response, but also the accessory role of Tfh and Th17 cells. It has been shown previously that Tfh cells are enriched in HIV-specific CD4+ T cells and serve as the major CD4+ T cell compartment for HIV infection (59), but these cells in some HIC/LTNP individuals appear to be more protected against infection and have a higher functionality to improve immune response.

In resting memory CD4+ T cell subset (CD45RO+CD25-CD69-HLA-DR-) a gene set enrichment approach showed an upregulation in immune response-related routes in EC, particularly those of T cell activation/functionality/proliferation and NK/NKT cell cytotoxicity/differentiation. On the other hand, the immune response pathways that were upregulated in TP-ART are mainly related to lymphocyte anergy, humoral, and mucosal immune response, in comparison to EC (60). Moreover, a meta-analysis of previously published datasets emphasizes the disparity in the regulation of the immune response between TP and LTNP. Altered pathways between both groups comprise overactivation of immune system process, response to virus stimulus, and inflammatory response (61). Another meta-analysis accounted for up to 14 common DEGs shared in comparisons of LTNP against healthy, TP and TP-ART patients, belonging to immune responses, antigen processing and presentation, T cell co-stimulation, and the interferon-gamma-mediated signaling pathway (62). Hence, we discern effective immune responses in HIV-resistant patients with polyfunctional and potent effects on infected cells and oppositely a sustained but vain immune response in TPs.

According to the knowledge obtained in PBMCs, IFN and ISG responses are markedly affected in CD4+ T cells in natural HIV resistance. There is a signature of upregulation of type I IFN-stimulated genes (*IFI16, IFI27, IFI44, IFI44L, IFITM3, MX1, OAS1, OAS2, OAS3*) associated to LTNP and non-HIV individuals, able to differentiate them from chronically and recently HIV-infected patients without treatment (63). However, CD4+ T cells from EC showed lower overall ISG activation compared to untreated TP, but similar ISG expression to TP-ART (64). Focusing on CD4+ T cells subpopulations, central memory (CD45RA-CCR7+CD27+) CD4+ T cells (TCM) from EC showed a decrease in the expression of genes implied in type-I IFN pathway (*IFI6, DDX21, IFIT1*) in comparison to naïve CD4+ T cells from the non-HIV group (65).

Concerning cell cycle, CD4+ T cells from HICs and LTNP display specific patterns in the regulation of pathways related to survival. Based on the enrichment of DEG in AKT (*PIK3R1, PIK3CA, PPP2CA, FOXO3A, FOXO1A, RELA, NFKB1A, NFKB1*) and WNT (*WNT1, WNT10A, WNT10B, FZD6, DVL3, TCF7, CTNNB1*) signaling pathways, CD4+ T cells from LTNP are supposed to be associated with better survival (56). However, a group of EC patients showed suppression of NF-kB signaling pathways caused by the downregulation of *HDAC6*, *ATM*, and *MAPK8* along with the upregulation of lncRNAs targeting these genes (66). Because NF-kB is a major transcription factor involved in HIV reactivation and immune activation, a reduced activity of this pathway could potentially decrease HIV replication through direct and indirect mechanisms. Similarly, the upregulation of PHLDA1 in LTNP may cause inhibition of AKT-signaling activation, a pathway that has been involved in the survival of infected cells, reactivation of latent provirus and HIV replication (62).

In like manner, cell surface receptor signaling pathways that are relevant for HIV replication repeatedly appear in these studies. For instance, MHC class II was downregulated in Gag-reactive HIV-specific CD4+ T cells from ECs and VCs when compared to chronic progressors (57), while T-cell receptor activation and costimulatory signaling pathways were affected in central memory CD4+ T cells from ECs (65). Another downregulation described in EC compared to TP involves *IL1R* and *IL6R*, as well as the upregulation of lncRNA that moderate their mRNA translation and lower inflammation levels (61). Besides, in the evaluation of the R5-tropic HIV resistance in activated CD4+ T cells from EC and VC, CD4+ T lymphocytes presented a downregulation of *CCR1, CCR2*, and *CCR5* transcripts (30). This downregulation could lead to a lower number of co-receptor molecules on the cell surface, thus hindering HIV entry, as it has already been proposed in the nonpathogenic infection of Simian Immunodeficiency Virus in their natural host (67).

Another biological process that is usually present in studies concerning HICs and LTNPs is related to cytoskeleton reorganization. Attending to two subgroups of EC identified by a principal component analysis, ECs that have an expression profile more similar to TP-ART show the upregulation of some genes from MAPK and focal adhesion pathways in comparison to ECs that group with non-HIV patients. These routes participate in cell cycling and cell migration (68). Similarly, in a meta-analysis of six transcriptomic data sets, *ACTB* and *ACTG1* were down-regulated in LTNP, which can modify the regular activity of the cytoskeleton and potentially tamper HIV-1 entrance, fusion, and production (62). The implication of these pathways might be related to HIV compartmentalization and systemic dissemination, and at cellular micro-level with the intracellular transport of HIV products.

Concerning post-transcriptional regulation by miRNA, transcriptomic studies in CD4+ T cells are scarce. The downregulation of the let-7 family of miRNA was described in TP and LTNP when compared to non-HIV patients. One of the experimentally validated targets of the let-7 family is IL-10, associated with immune response impairment and viral persistence in chronic infections. Measuring IL-10 levels in those patients whose let-7 members were downregulated showed that TP had significantly higher expression levels than HIV-negative controls, while LTNP presented intermediate levels. This correlates with the let-7 family members’ expression level in all groups, suggesting that CD4+ IL-10 production is governed by let-7 miRNAs thus conferring a better clinical response in LTNP lowering IL-10 levels (69).

#### 2.2.2 CD8+ T Cells

CD8+ T cells are key in adaptive immune response and important for viral clearance during HIV infection. Transcriptomic studies have also described that MAPK, WNT, and AKT pathways in CD8+ T cells are associated with HIC and LTNP individuals. PI3K-AKT pathway is upregulated in ECs (70), and genes associated with AKT activation (*PIK3R1, PIK3CA, and PPP2CA*) were upregulated in LTNPs (56), in comparison to HIV viremic progressors. Likewise, eIF2 and mTOR signaling cascades, proteasome, the interferon-induced immunoproteasome, cell cycle, cell division, and metabolic pathways may play an important role in the regulation of CD8+ T cell function in ECs (5, 70).

Similar to CD4+ T cells, several works showed that ISGs (*IFI44, MX1, EPSTI1, PARP12*, among others) are downregulated in LTNP/HIC individuals compared to HIV progressors (5, 56, 63, 70, 71). Other DEGs have also been found in individual works. For example, *BATF* is downregulated in exhausted HIV-specific CD8+ T cells from HICs (71); *BAG3* is upregulated in LTNPs (56); *ATP6V1D, ACTA2, PSMB2* and *PSMA5* are downregulated in LTNPs (56).

Furthermore, as it is mentioned above, there could be differences in gene expression between subgroups of HICs. Comparing CD8+ T cells from strong responder HICs and weak responder HICs, defined by high and low production of TNF-α, IFN-γ, and MIP-1 respectively, genes involved in IFN-γ pathway were up-regulated in strong responder HIC, whereas pro-inflammatory genes (*CXCL8, IL1B, IRAK3, TYROBP*, and *FCER1G*) were downregulated in these individuals. Likewise, strong responders HICs also exhibited a significant upregulation of *CX3CR1*, a marker of CD8+ effector memory cells. Among specific routes enriched in DEGs between CD8+ T cells from weak responder HIC and strong responder HIC, four main functions were identified: T cell response, cytotoxicity of leukocytes, killing NK cells, and activation of leukocytes (45).

Recent transcriptome analyses have revealed different gene expression levels in CD8+ T cell subpopulations from HIV-infected people. One of them described a higher frequency of CD8+ T resident memory cells (TRMs) in lymph nodes from ECs. HIV-specific CD8+ TRMs showed lower levels of *S1PR1, FLF1*, and *SELL* genes, and are enriched in effector-related immune genes compared to HIV-specific non-TRMs. Also, single-cell transcriptomic analysis on CD8+ TRM cells of lymph nodes from ECs revealed differences in cell location pathways, but not in cytolytic activity, compared to this cell type from blood (72).

Another work reported CD8+ T cells with distinct functional, phenotypic, and transcriptional signatures, including the upregulation of soluble factors, efficient protein translation, and high ribosome biogenesis. This gene expression profile is thereby associated with the ability to suppress HIV replication in lymph nodes from ECs. These HIV-specific CD8+ T cells showed a non-cytolytic feature and expressed lower levels of *PRF1* and *GZMB* in lymph node from ECs, as well as preferentially expressed *IL7R* (closely related to T cell homeostasis), *CCL5*, and *IL32* genes (related to highly functional memory phenotype and suppression of HIV replication) (73).

As in other populations, CD8+ T cells’ miRNA expression profile has been assessed. Several miRNAs were DE comparing ECs and VCs to HIV uninfected individuals, and regulation of signal transduction and metabolic regulation are the most related pathways associated with this miRNA profile. The most downregulated miRNAs in ECs are miR-3665 and miR-2861, while hsa-miR-150 and hsa-miR-1202 are the most repressed in VCs, after *in vitro* CD8+ stimulation (74).

### 2.3 Other Immune Cells

Transcriptomic profiling of cells actively participating during HIV infection such as monocytes and DC have also been performed. Focusing on ISG expression in CD14+ primary monocytes, *LY6E* downregulation is inversely correlated to CD4+ T cell counts and CD38+CD8+ T cells frequency in LTNPs (75). Analyzing monocytes from another cohort of LTNPs, a higher number of DEG was observed when compared with HIV-infected individuals with a low viral load. Also, monocytes from LTNPs showed some DEG-enriched pathways that are involved in cell signaling, cell cycle, apoptosis, metabolism, cytoskeleton, cell migration, and phagocytosis, as well as a higher expression of *TLR4, TLR8, IL1B* and/or *IL8*, which are related to TLR signaling and cytokine-cytokine receptor interaction pathways (76). Another transcriptomic analysis of monocytes from neutralizer ECs showed antigen presentation, immune regulation, and cytokine-dependent cell interaction as the top pathways correlated to neutralizing antibody breadth (58). Some of the molecular cascades described in monocytes have also been found in DCs from HICs. Martin-Gayo et al. revealed remarkable functional heterogeneity among myeloid DC (mDC) (CD14-, CD11cHi, HLA-DR+), described by several discrete transcriptional response states after HIV infection. A subset of mDCs expressed a specific transcriptomic profile in a cell cluster related to a better immune response. These cells (c1 mDCs) from EC displayed a thorough functional activation, higher innate antiviral activity, and a more efficient induction after *in vitro* viral exposure, in comparison to HIV-1 chronic progressors. Some genes as *IFIT3, FCGR1A, IFI6*, including other ISGs, are overexpressed in c1 mDCs compared to the rest of mDC subsets, as well as innate recognition, DC maturation, IFN and TLR signaling pathways are overrepresented in these cells (77).

Additionally, another report has described a different transcriptional pattern and specific phenotypic features of an mDC subset from HICs. Comparing transcriptomic data of mDCs from neutralizers (Nts) and non-neutralizers EC (NNs), there is a subgroup of Nts (Nt2) associated with an enrichment of transcripts related to T cell co-stimulation (*CD40, CD28, ICOS*), improved B cell receptor signaling, and activation of cytokine signaling. This pattern of expression suggests an enhanced functional state of Nt2 mDCs. Also, these cells expressed significantly higher levels of co-stimulatory molecules such as *CD83, CD86, PD-L1, PD-L2*, and *CD40* (58).

### 2.4 Immune Cell Dynamics: Integrating Transcriptomic Data

Some meta-analyses analyzed transcriptomic data of whole blood, CD4+ and CD8+ T cells from different studies. These transcriptome meta-analyses have identified a common reduced expression of several ISGs (*ISG15, LY6E, MX1, OAS1, OAS2, OAS3, OTOF, RNASE2, USP18, XAF1*) in T cells from LTNP/HICs compared to HIV progressors (78, 79). Additionally, expression of *CMPK1, CBX7, EIF3L, EIF4A* and *ZNF395* were highly correlated with viremic control (79).

Zhang et al. describe an upregulation of several genes as *CCR7, NELL2, MMD, SORL1, FAM46C*, and *RBM38*, among others, as well as an upregulation of cytokine–cytokine receptor interaction (CD4+ T cells) and MAPK signaling pathway (CD8+ T cells) (78).

A recent work performed integrative analysis between transcriptomic profiles of different immune cell populations from HICs using RNA-seq data. Martin-Gayo et al. analyzed several cell populations and suggested the existence of multi-compartment immune networks between mDCs, Tfh, and monocytes, which may facilitate the development of broadly neutralizing antibodies in a group of HICs. A functional computational analysis of each gene module indicated significant reciprocal associations between immune recognition and immune activation pathways in mDCs, CD4+ T cells, and monocytes from Nt2 (a subgroup of ECs) patients, indicating close connections between individual functional modules from these different cells compartments (58).

Although meta-analyses approximation is useful for the discovery and confirmation of natural mechanisms of resistance, we must notice the limitation associated with the selection of datasets based on heterogeneous definitions of HIC/LTNPs.

### 2.5 Common DEGs and Biological Routes Expressed in LTNP/HIC Individuals

In this section, we highlighted the common DEG among different immune cell populations. Early transcriptome analyses in CD4+ and CD8+ T cells showed that ISGs are the main DEG set between LTNP and HIV progressors (63). Besides, response to stimuli and signal transducer activity pathways were significantly enriched in LTNPs, and Rho signaling pathway on HIV viremic progressors (56). Also, genes associated with AKT (*PIK3R1, PIK3CA*, and *PPP2CA*) and MAPK signaling pathways (*ERK, JNK*, and *P38*) are upregulated in LTNP individuals, which could contribute to cell survival and stronger anti-HIV responses (56).

On the other hand, taking into consideration results from different transcriptomic studies, most of them have shown a differential expression of ISGs and several genes involved in inflammation, proliferation, and/or activation pathways, among others, in LTNP/HIC individuals compared to HIV progressors. Several studies have repeatedly reported a common downregulation of several ISGs: *ISG15, LY6E, MX1, OAS1, OAS2, OAS3, OTOF, RNASE2, USP18*, and *XAF1* in monocytes, and CD4+ and CD8+ T cells from LTNPs and/or HICs in comparison with non-controllers and TPs (5, 62, 71, 76, 78, 79). Although the implication of ISG is notorious, it is important to consider the clinical/control groups included in the studies, since the direction of gene expression may vary according to the reference group. The recurrent deregulation of IFN genes and ISGs highlights its importance during HIV infection. It is expected that HIV-infected patients after ART can downregulate these sets of genes to the levels observed in LTNP and EC individuals thus avoiding the underlying persistent activation of ISG that contributes to chronic inflammation. However, certain ISGs have a higher expression in LTNP. For instance, we observed that *IFI27* and *IFITM3* have higher expression in CD4 and PBMCs from LTNP (44, 63) which could be related to a beneficial effect of immune activation against HIV in these individuals.

Previous works on PBMCs have reported upregulation of genes associated with the control of activation, proliferation, inhibition of apoptosis, and survival of T cells (*HSH2D, STAT5B*) or genes related to the regulation of acute inflammatory response (*CCR2, C5AR1, C3AR1, SERPING1*) in LTNP individuals (44, 48). Similarly, in specific immune populations (monocytes, CD4+ T cells and/or CD8+ T cells) from LTNP/HIC individuals, a differential regulation of genes implicated in the activation (5, 45, 60, 63), proliferation (5, 60), apoptotic processes (47, 76), inflammation (45) and cell survival (56, 62) responses has been described.

Moreover, transcriptomic studies have revealed a relationship between calcium metabolism and HIC/LTNP individuals. Previous findings have reported upregulation of genes (*NTSR1, FPR1, ITPR3, CALM-1*) and cell pathways (PI3K-AKT pathway) related to uptake, signaling, transport, or mobilization of calcium in T cells and PBMCs from HIC/LTNPs (44, 48, 62, 70, 78). Ca2+ can act as a second cell messenger in lymphocytes, which at resting state maintain low Ca2+ concentration and increase after various antigenic stimuli to fulfill their immune defense functions (80), playing an important role in HIV control.

Other commonly identified DEGs are also related to the repression of viral transcription. Several transcriptomic analyses showed upregulation of *GADD45* family, *IER3*, and/or *CDKN1A* in different cell types (PBMCs, T cells, and monocytes) from HICs and LTNPs, in comparison to HIV progressors (44, 71, 78, 79). In addition, *CDKN1A* is upregulated in EC-LTNP, promoting the arrest of the cell cycle, and its upregulation is correlated with *TNF, IER3*, and *GADD45B* expression. These genes coordinately interact with 13 proteins and polyproteins of HIV-1, inhibiting basal and Tat-dependent HIV-1 transcription (44).

Additionally, some genes of the SERPIN family (*SERPINB2, SERPING1*) are overexpressed in monocytes and PBMCs from LTNP individuals. Besides*, CMPK, XCRR*, and *HERC* families have a differential expression in T cells and PBMCs from LTNP and/or ECs compared to HIV progressors (44, 62, 76, 78, 79). Although the role of some of these genes in HIV replication of infected cells has been proposed, their role in non-HIV-infected cell types (e.g. CD8+ T cells) remains uncertain.

**Figure 1:**
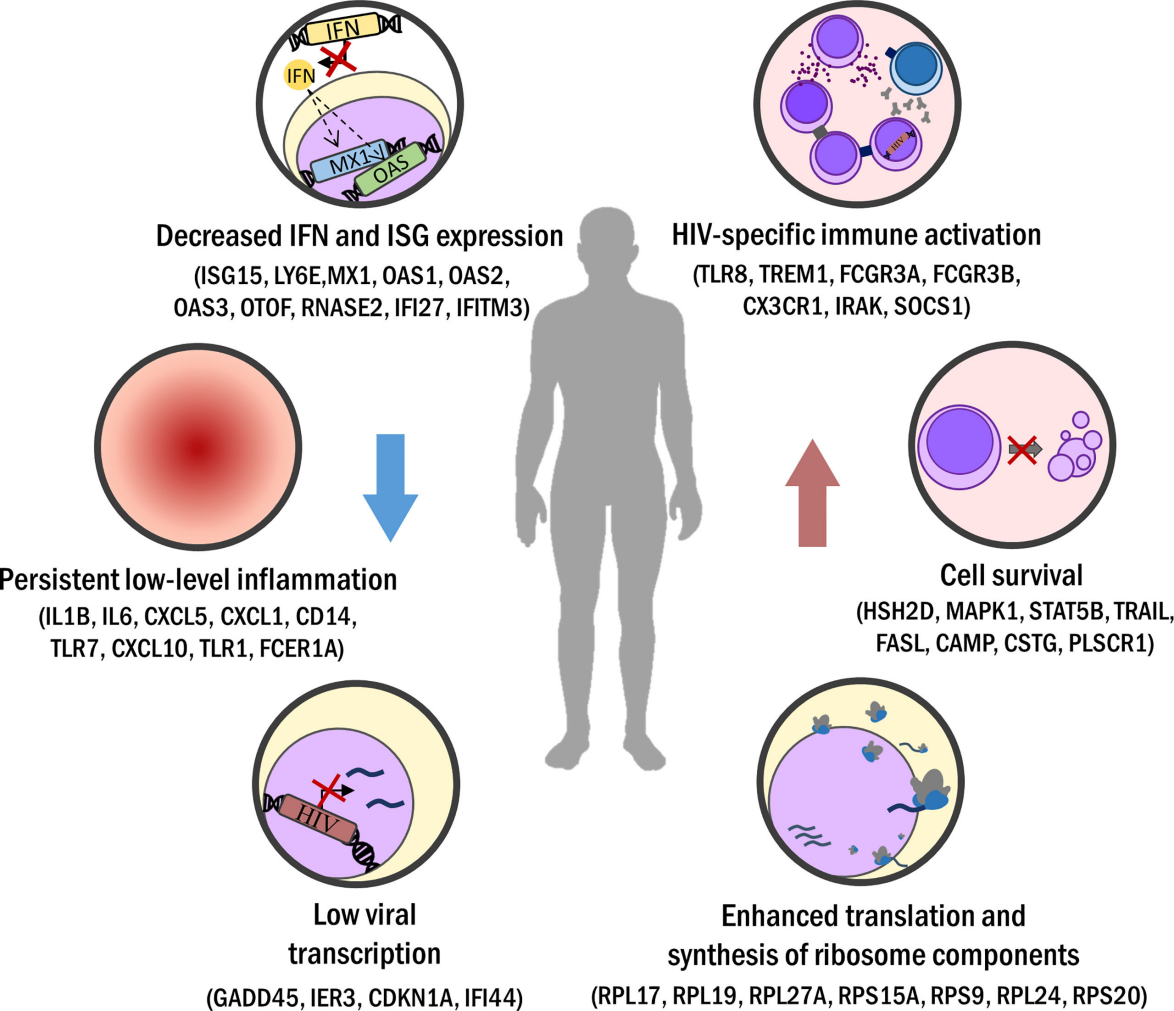
Biological processes commonly seen in elite control or long-term non-progression. Minor activation of sustained immune response (i.e. downregulated IFN response) leads to persistent but low inflammation levels in EC/LTNP. Viral transcription is lessened in EC but also in LTNP. Increased HIV-specific cellular immune response is registered, driven by a strong Th1 CD4+ T helper activity and enhanced CD8+ T cells cytotoxicity and polyfunctionality. Apoptosis hindrance and favored expression of cell cycling pathways improve cell survival. Also, ribosome component amount and protein synthesis are increased in EC/LTNP.

Also, several works have shown an overexpression of genes or pseudogenes related to ribosome components (*RPL17, RPL19, RPL27A, RPS15A, RPS9, RPL24, RPS20, RPS28, RPL36, RPL9, RPL31, RPL5P4*, among others) in T cells and PBMCs from HIC/LTNPs (44, 70, 79). Therefore, gene expression of ribosome components appears to play an important role in the progression and/or control of HIV, but the mechanisms involved in these processes remain to be defined. Considering several studies, we may conclude that it is difficult to find a common transcriptomic signature among HIC/LTNP individuals. However, there are some repeated patterns, as summarized in **Figure 1**, depending on the analyzed HIV-infected phenotype and the control group. For instance, it is not usual to find DEG comparing ECs or LTNP to HIV-negative individuals (63, 64, 70, 81). Additionally, HIV-positive patients under successful ART also present similar profiles to HIC/LTNP patients in some studies (56, 64).

## 3 Discussion

### 3.1 Transcriptomic Technologies: RNA-Seq or Arrays

Since the late 90s, transcriptomic profiling was based on hybridization technologies. Affymetrix and Agilent microarrays gained a lot of popularity before the appearance of high-throughput sequencing techniques, classically carried out by the more time-cost Sanger sequencing coupled with tagging methods. Thus, gene expression microarrays were established as the tool of preference for transcriptomic assessment during a whole decade (82–84). Hence, a great part of transcriptomic studies in LTNP, HIC, or EC is conducted with microarrays platforms. However, the advantages of RNA-Seq outweigh a few aspects of microarray technology. RNA-Seq provides a wider quantitative range of expression level changes, especially in transcripts expressed at low levels, and therefore works with less quantity of starting RNA. It can identify alternatively spliced transcripts, isoforms, indels, and SNPs with more accuracy; detects non-coding regions usually not included in microarrays, and also register any part of a whole-genome while array technologies rely on the designed probes included on the chip (85). Besides, the drawbacks of RNA-Seq as, larger output files and the requirement of bioinformatics skills are being overcome by the continuous lowering of sequencing cost, the fabrication of platforms with higher throughput capacities, and the development of friendlier software for the analysis of the results (86). On the other hand, the standardization of sample preparation protocols and downstream analysis for RNA-Seq projects is a remaining task (87). On those grounds, it is expectable that RNA-Seq technology will replace microarray platforms in transcriptomic studies, as the problems of high cost, storage, and data analysis are solved.

### 3.2 RNA-Seq for the Identification of Genetic Variants

The RNA-Seq technology has also potential for the detection of genetic variants in the host genome. Whole-genome sequencing comprises the polymorphisms of the full-length genome but is a high-cost technique. On the other hand, the sequencing of gene-coding sites like Whole Exome Sequencing or RNA-Seq reduces the economical and computational costs versus whole-genome sequencing. This fact underlines RNA-Seq as a good choice for sample genotyping at the same time as transcriptome assessment. Nissen et al. performed Whole Exome Sequencing on ECs and LTNPs and they observed low-frequency variants were in genes involved in innate immune response (*LRRIF1P, IRAK2, TAB2, NOD2, SLX4*), HIV transport (*FN1, DDOST, PRKCA, FRK*, among others), and HIV transcription (*MED6, PRKDC, CCNT1, EGF*) mainly within the LTNP group. Also, LTNP with genetic variants within *TLR* and *NOD2* pathways presented reduced pro-inflammatory responses compared to controls (88). RNA-seq can be used for the detection of genetic variants within the transcriptionally active region of the genome. In this alternative approach, RNA molecules transcribed only from exons are sequenced with enough depth to identify genetic polymorphisms. The advantages compared to Whole Exome Sequencing are lower cost, lower storage, and data analysis requirements while focusing upon expressed coding regions of a given sample (89). This also leads to a loss of information of the coding regions that are not expressed in the tested tissue/cell type and in non-transcribed and regulatory zones, but to our knowledge, it is a reliable method for the detection of nucleotide variants associated with a better response to HIV infection.

### 3.3 Single Cell Transcriptomics

Single-cell technique for transcriptome analyses of HIV-infected cells has also been used. A work performed in a primary cell model of HIV latency has allowed the identification of transcriptional heterogeneity of latently infected cells and viral reactivated cells after latency reversal agents stimulation. Also, 134 DEGs were found in common between cell clusters after latency reversal agents stimulation, and they were involved in processes related to the metabolism of RNA and regulation of protein, electron transport, RNA splicing, immune system, HIV infection, and translation (90). Another work used a cell model of HIV latency to investigate viral RNA expression and the role of the host transcriptome on HIV restriction. They showed that HIV downregulation is associated with the expression of a specific transcriptional signature related to markers of naive and central memory T cells. Cells with undetectable HIV expression showed a higher expression of *CCR7, CXCR4*, and *SELL* genes (38). Both approaches reveal that the host cell transcriptional program is related to the expression of HIV proviruses into the latent reservoir, and together can be modified by cell stimulation. The single-cell sequencing approach has also gained relevance in the determination of the genetics of specific immune cells directly involved in better viral control and/or non-progression. According to this fact, some works have been performed in CD8+ T cells and mDCs from HICs, determining differences in the transcriptomic profile of these cell populations, as well as an association to a better immune response against HIV and/or specific phenotypic features (72, 77).

However, the assessment of single-cell transcriptome of CD4+ T cells from LTNP/HICs is troublesome because integration, latency, and low-level replication of HIV could modify cellular gene expression. In addition, current technologies have technical limitations to differentiate between HIV-infected and uninfected cells, without gene expression´s meddling. The improvement and application of these techniques in immune cells subpopulations would allow us to perform a unique experiment that correlates cell gene expression with proviral deletions or provirus integrations sites, as well as the identification of surrogate markers of different states of HIV infection.

### 3.4 Source of Variability in Results

Experimental procedures could be important sources of variability. The type of sample, time and conditions of storage, RNA extraction technique, and sample preparation for transcriptomic platforms are quite different in the studies analyzed in this review. Also, participants’ medical records such as their lifestyle are also covariates to consider in the design of transcriptomic studies. Despite that data normalization and batch-effect correction algorithms are applied to avoid these confounding factors (77, 91), it is clear that a high number of replicates is the best way to capture the most accurate representation of our study population.

Besides, the phenotypic definition in HIV control is often redundant as well as imprecise, while also caused by multiple actors as virological, genetic, and immunological factors (2). Moreover, phenotypical comparisons between HIV-infected individuals render distinct DEG attending to the samples used as controls, which are usually HIV-negative individuals or HIV typical progressors on ART or without treatment. Selecting the control population for transcriptomic studies is crucial and each of mentioned phenotypes could offer different advantages in the search of genetic markers. For instance, comparing phenotypes with low or undetectable VL, as HIV typical progressors receiving ART and EC individuals, would allow us to find genes associated with natural viral control. However, if we compare HIV typical progressors in absence of ART with viremic LTNP, we could find a gene expression profile that avoids immune cell loss in non-progressor individuals.

In the same manner, the lack of consistency in clinical definitions and experimental designs may hinder the performance of conclusive meta-analyses. This situation establishes the necessity of normalizing the study procedures. Lastly, we must consider that transcriptomic profiles could be dynamic during HIV infection phases, and a certain range of variation in gene expression level is expectable between different studies.

Diverse and complex gene-expression profiles have been observed for equivalent samples from LTNP/HIC phenotypes. Although we might neglect underrepresented mechanisms participating in HIV control, the results obtained in transcriptomic projects are robust in each work. Indeed, we have shown common regulated genes and altered pathways that appear regularly in transcriptomic analysis.

However, DEG and biological routes found in some studies are not always identified in other analyses. This could be due to different technological platforms, diverse bioinformatics tools to analyze gene expression, or the features of patients, as mentioned above. For these reasons, stricter and more homogeneous phenotypical definitions should be developed to conduct meta-analyses of relevance.

## 4 Future Directions

### 4.1 Integration of Omic Data From Different Technologies in the Study of HIC/LTNPs

In recent years, different meta-analyses have been performed in LTNP/HICs, using mainly genomic or transcriptomic data from different populations, as an advantageous approach to find common markers related to HIV control or non-progression as we described above. Likewise, integrative analysis that includes transcriptomic, genomic, proteomic and/or epigenomic data, among other –omic data, together with immunological parameters, could be a powerful tool to discover biological mechanisms that involve a global immune response of HIC/LTNP individuals. For instance, a parallel analysis of mRNA and miRNA transcriptomic profiles showed that neurodegenerative processes in HIV disease are linked to biological routes and biomarkers associated with axon guidance and its downstream signaling pathways (92).

Some studies have measured the gene expression profiles of CD8+ T cells, CD4+ T cells, and/or monocytes from HIC/LTNPs and correlated them to some immunological markers (5, 45). However, integrative works that unify immunological and transcriptomic data from immune cell subpopulations are scarce. Some recent studies associate immunological markers from specific cells subpopulations involved in HIV infection with specific gene expression profiles, indicating that effector immune response, antiviral activity, and/or cell location pathways are related to HIV control in HIC individuals (58, 72, 73, 77). Future studies in CD4+ or CD8+ T cells should be performed in a similar way, due that only a few transcriptomic studies of LTNP/HICs have included immunological parameters in their analyses. This could allow us to determine a better association between transcriptomic profile and immune cells clusters immunophenotypically defined.

On the other hand, transcriptomic profiles can be different in a specific immune cell population of HICs from the same cohort (68, 70). Gene expression could be influenced by genetic or epigenetic factors, but these interactions have not been elucidated precisely in HIC/LTNP individuals. RNA-seq data could be used to search rare or less frequent genomic variants into gene coding regions, associated with its gene expression profile, which is related to control and/or non-progression. Also, the generation of new RNA-seq data in immune cell populations from HIC/LTNPs could allow us to perform meta-analyses to search for common genes associated with these HIV phenotypes. Thus, it would be necessary to implement or improve pipelines to analyze these bioinformatics approaches.

Along with differential expression analyses between phenotypes, hierarchical clustering is another informative way to analyze transcriptome data. To date, it has been used to classify LTNP/HIC individuals and HIV progressors (45, 63). However, there is not any set of DEGs able to predict LTNP/HIC phenotype with high accuracy. To achieve that, machine-learning techniques could be a good approach to search for a set of genes able to predict phenotypes of HIV infection. For instance, our research group described a set of 20 genetic elements (13 genes, 6 pseudogenes, and 1 long non-coding RNA), whose level of expression classifies patients according to their progression state (LTNP or TP) with a 90% of accuracy (44). The studies to come should include similar analyses to find common features to identify predictive biomarkers or correctly classify EC/LTNP patients, including host genomic variants, immunological, and/or virological data, to perform an integrative analysis and to consider a bigger picture.

### 4.2 Spatial Transcriptomics: Describing Tissue-Related Signatures

The spatial distribution of cell types molecularly defined by their transcriptomic profiles can be characterized by a still evolving technology referred to as spatial transcriptomics. Mapping gene activity in relative locations within tissue compartments is critical to understanding the anatomical perspective of the effective immune control observed in HIV controller and LTNP phenotypes. The main target for these studies are lymphoid tissues as major reservoir sites for HIV. Although spatial transcriptomics is advancing rapidly thanks to new *in situ* RNA sequencing and multiplex fluorescence *in situ* hybridization (FISH) technologies, some issues need to be addressed in the near future, such as the cost or throughput capacity as well as the standardization of analysis methods (93–96). The technologies to explore the anatomical compartmentalization of HIV-1 reservoir in tissues by spatial transcriptomics has to be at single-cell level, due to the extremely limited pool of HIV-1+ cells observed in EC-LTNP (20), and has to simultaneously assay the proviral DNA integration and viral gene expression (97, 98). In this sense, the study of HIV-1 integration landscape adds a new level of complexity in the analysis by requiring the combination of other technologies such as single cell RNA-Seq with ATAC-Seq and Hi-C-Seq to assay chromatin accessibility and topologic domains, MIP-Seq for the simultaneous sequencing of host integration sites and proviral intactness, and ChIP-Seq and bisulfite-seq to identify epigenomic signals in histones and DNA methylation in the integration sites (20, 99). The integrative omics in tissue compartments beyond blood will be critical to advancing our understanding of the underlying mechanisms of HIV control and disease progression observed in elite controllers and LTNP.

### 4.3 Finding Targets for Treatment and Functional Cure

Transcriptomic analyses provide data that could be useful to improve current HIV therapy or to evaluate specific surrogate or predictor markers of efficacy in particular immune cell populations after immunomodulatory interventions directed to achieve the HIV functional cure. Quigley et al. observed a higher *BATF* expression in HIV progressors in comparison to HICs. Additionally, the performed *in vitro* functional experiments showed that positive regulation of *BATF* is associated to a higher *PD-1* expression, which inhibits the function of HIV-specific CD8+ T cells, as cell proliferation and cytokine secretion (71). Drugs able to counteract these effects could improve immune recovery and control HIV replication.

On the other hand, some of these studies have been useful to propose potential biomarkers of HIV progression. For instance, low levels of miR-630 could be a prognostic marker of disease progression in LTNP (55) and low levels of *LY6E* in the absence of ART, which correlates with functional and competent immune response, can serve as a biomarker of LTNP condition (75). However, the achievement of spontaneous control of HIV infection by LTNP/HIC individuals cannot be explained by the presence of a single factor, but rather has a multifactorial nature (100). It has been proposed that EC/LTNP individuals with several ‘protective’ HLA alleles, stable levels of CD4+ T cells, prolonged viral control, and absence of virus evolution, may have achieved a functional cure of HIV (101), one of the main goals of current HIV research. For this purpose, omic technologies could be a powerful tool to identify biological markers able to predict or evaluate therapeutic interventions aimed to achieve HIV functional cure. For instance, Perera et al. monitored disease progression during 20 years after HIV p24 vaccination in an EC patient, through proteomic analysis. They observed the upregulation of CXC3R1, ITAC, IGFBP-2, TECK, TRAIL-R4, and MIP-1α in CD4+ and CD8+ T cells and monocytes during the aviremic phase of follow-up, indicating a potential role in viral control during HIV-1 infection (102). On the other hand, whole-exome sequencing has allowed the identification of variants in the *CNOT1* gene as potential candidates to predict the response to treatment with therapeutic vaccines based on DCs (103). Longitudinal studies are needed to evaluate the success of therapy in an HIV-infected patient, or the changes associated with the loss or the maintenance of viral control in LTNP/HICs. For this purpose, RNA-seq technology could offer us a powerful tool to pursue these goals. Additionally, data analysis could be improved to search and correlate both gene expression and genetic variants in a single experiment.

Current approaches highlight that both induction of HIV-1-specific helper cells and coordinated cellular immune response might be important in immunotherapeutic interventions and HIV vaccine development. These new immunomodulatory approaches should lead to strong HIV-1-specific immune responses while minimizing inflammation, which probably allows controlling VL below detection limits and/or maintaining high levels of CD4+ T cell counts, like HIC/LTNP individuals. Also, future omic analyses of specific immune cell populations could contribute to the search of biomarkers of long-term remission or functional cure from HIV-infected people under these new therapeutic approaches. For instance, single-cell transcriptome sequencing in HIV-infected cells would be useful to search specific gene expression profiles related to the maintenance or reversion of viral latency, allowing us to design better strategies to decrease or eradicate the HIV reservoir.

## Author Contributions

All the authors contributed to the article’s conception. ET-T and RA-S performed literature research and drafted the first manuscript of the work. FD-F and JA critically revised the manuscript and approved the final version. All authors contributed to the article and approved the submitted version.

## Funding

This study was funded by a fellowship from GILEAD Sciences (GLD18/00090), Instituto de Salud Carlos III (PI19CIII/00004), and has been conducted within the Spanish AIDS Research Network (RIS), funded by Instituto de Salud Carlos III (Plan Estatal de I+D+I 2013-2016) and co-funded by European Regional Development Fund (ERDF) “A way to build Europe’’ (RD16CIII/0002/0001). RA-S was supported by the Ministry of Innovation, Science and Universities predoctoral funding (FPU18/05527).

## Conflict of Interest

The authors declare that the research was conducted in the absence of any commercial or financial relationships that could be construed as a potential conflict of interest.

## Publisher’s Note

All claims expressed in this article are solely those of the authors and do not necessarily represent those of their affiliated organizations, or those of the publisher, the editors and the reviewers. Any product that may be evaluated in this article, or claim that may be made by its manufacturer, is not guaranteed or endorsed by the publisher.
