# Supplementary material for: Omic Technologies in HIV: Searching Transcriptional Signatures Involved in Long-Term Non-Progressor and HIV Controller Phenotypes
Source: Front Immunol. 2022 Jul 1;13:926499. doi: 10.3389/fimmu.2022.926499 (PMC9284212; doi:10.3389/fimmu.2022.926499)
Supplement: Supplementary file 1 [file Table_1.docx]

Supplementary Material

**Supplementary Table 1. Features of transcriptomic researches: Analysis platform, sample size, clinical definitions and cell populations of study.**

| **Technology** | **Populations** | **Definition of LTNP and HIC individuals** | **Cell types** | **Reference** |
| --- | --- | --- | --- | --- |
| Microarray | LNTP (n=5), HIV negative (n=5), HIV progressors in absence of ART: chronic (n=5) and early (n=5) infection | LTNP: follow up > 3,5 yrs (80% > 9 yrs); no CD4+ T cell decline; VL < 500 (<269 - <50) | CD4+ T cells,  CD8+ T cells | Hyrzca et al, 2007 – (63) |
| Microarray | EC (n=16), HIV negative (n=3), HIV progressors (n=137) | EC: Asymptomatic > 10 yrs from seroconversion. VL < detection limit in 3 consecutives samples within a year (Isolated blips < 1000 cp/ml allowed) | CD4+ T cells | Rotger et al, 2010 – (64) |
| Microarray | HICs: ECs (n=20), VCs (n=4); HIV progressors in absence of ART (n=18) | HICs: follow up 7-20 yrs; CD4+: 792 (666-945); EC: <75; VC: <2000 | CD8+ T cells | Quigley et al, 2010 – (71) |
| Microarray | LTNP (n=11), HIV progressors in the absence of ART (n=13) | LTNP: follow up > 10 yrs; CD4+ > 500; VL < 10 000; | CD3+ T cells | Salgado et al, 2011 – (55) |
| Microarray | Elite/viremic controllers (EC/VCs) (n=9), HIV progressors in absence of ART (n=5), viremic non progressors (VNPs) (n=6) | EC/VC: follow up >10 yrs ; CD4+: 858 (655-1140); EC: VL <75 (with isolated viral blips up to 1000); VC: VL <2000. VNP: VL > 300,000 (> 3 determinations), CD4+ > 350; follow up > 3 yrs | CD4+ T cells, CD8+ T cells | Rotger et al, 2011 – (5) |
| Microarray | EC (n=12), HIV progressors on ART (n=14), HIV negative (n=9) | EC: Stable CD4+ levels up to 10 yrs. Undetectable VL by commercial assays. | CD4+ T cells | Vigneault et al, 2011 – (68) |
| Microarray | LTNP (n=4), HIV progressors on ART with detectable VL(n=5) and undetectable VL (n=5); HIV negative (n=5) | LTNP: follow up >14 yrs; CD4+ > 500; VL < 50; | Whole Blood, PBMCs, CD4+ and CD8+ T cells | Wu, 2011 – (56) |
| Microarray | HIV progressors on ART (n=7), LTNP(n=7), HIV negative (n=8) | LTNP: VL < 300; VL > 4 500 | CD4+ T cells | Swaminathan et al., 2012 – (69) |
| Microarray | HIV negative (n=8), EC (n=7), HIV progressors in absence of ART (n=7) | EC: VL <50 | PBMCs | Witwer, 2012 – (52) |
| Microarray | LTNPs (n=5), HIV progressors on ART: VL>1000 (n=5) or VL<1000 (n=5), HIV negative (n=5) | LTNP: follow up >20 yrs (80%); VL <57 (80% <40); CD4+ >500 | Monocytes | Wu et al, 2013 – (76) |
| Microarray | viremic non progressors (VNPs) (n=9) and HIV progressors in the absence of ART (n=10) with early infection | VNP: CD4+ > 500 (>15% of PBMC); VL > 10 000; | CD4+ (subpopulations) | Klatt et al, 2014 – (46) |
| Microarray | EC-LTNP (n=7), vLTNP (n=7), HIV negative (n=7) | EC-LTNP: VL 60 - 1960; vLTNP: VL 934 - 54000 | CD4+ (subpopulations) | De Masson et al, 2014 – (65) |
| Microarray | HIV non-progressors (n=15), HIV progressors in the absence of ART (n=11) | HIV non-progressors: VL <5000 | PBMCs | Xu et al., 2014 – (75) |
| Microarray | LTNP (n=9), HIV progressors on ART (n=6), HIV negative (n=8) | LTNP: CD4+ >500; VL < 10 000 in all determinations | PBMCs | Luque et al., 2014 – (48) |
| LNA qPCR array | HIV negative (n=6), HIV progressors on ART (n=9), EC (n=9) | EC: VL <50 (1 yr); CD4+ > 350 (last 5 yrs) | Plasma | Reynoso et al, 2014 – (51) |
| TaqMan Array | HIV negative (n=5), EC (n=9), HIV progressors in absence of ART (n=8), HIV progressors on ART (n=8) | EC: follow up > 6 yrs; VL < 50; CD4+ > 450 | PBMCs | Egaña-Gorroño et al, 2014 – (53) |
| Microarray | HIV negative (n = 11), EC (n = 15), VC (n = 15), VP (n = 13); HIV progressors on ART (n = 14) | EC: follow up > 1 yr; VL <50; VC: VL <2000; CD4+ > 450 | CD8+ T cells | Egaña-Gorroño et al, 2016 – (74) |
| Integrative Meta- analysis: Microarray | LTNP (n=12), HIV progressors (n=12), HIV negative (n=13). | Transcriptome data: obtained from Hyrcza, 2007; miRNome data: obtained from Swaminathan, 2012. | CD4+ T cells | Liao et al, 2017 – (61) |
| Integrative Meta- analysis: Microarray | LTNPs & ECs (n=81), control group (Rapid progressors + HIV progressors)(n=98). | Those used in Hyrcza, 2007; Quigley, 2010; Rotger, 2011; Klatt, 2014 and Xu, 2014. | Whole Blood, CD4+ and CD8+ T cells | Zhang et al, 2017 – (78) |
| Microarray | EC (n=51), HIV progressors on ART (n=32) , HIV negative (n=10) | EC: follow up ≥ 2 yrs; VL <50 or <75 (≥ 2 consecutive determinations) | CD8+ T cells | Chowdhury et al., 2018 – (70) |
| RNA-seq | ECs (n=9), HIV progressors in the absence of ART (acute and chronic stages) (n=44), HIV progressors on ART (n=19), HIV negative (n=51) | EC: VL <40; CD4+: 835 (643- 1111) | CD8+ T cells | Buggert et al., 2018 – (72) |
| RNA-seq | ECs (n=8), HIV negative (n=7), HIV chronic progressors (n=8) | EC: follow up median 5 yrs (2–14); VL 20 - 98; CD4+: 909 | mDCs | Martín-Gayo et al, 2018 – (77) |
| RNA-seq | EC (n=19), HIV progressors in absence of ART (n=8), HIV negative (n=14) | EC: follow up > 1 yr; VL < 75 (one blip allowed always < 1000) in > 3 consecutive determinations. Also HIV+ > 10 yrs with >90% VL < 400. | PBMCs | Zhang et al., 2018 – (49) |
| RNA-seq | ECs (n=12), HIV progressors in absence of ART (acute and chronic stages) (n=25), HIV progressors on ART (n=14) | EC: VL<40, CD4+: 903 (657-1542); follow up 198 months (120-321) | CD8+ T cells | Nguyen et al., 2019 – (73) |
| Microarray | HICs (n=53): 10 strong responder HICs and 9 weak responder HICs; HIV progressors on ART (n = 27) | HIC: VL <400; CD4+: 689 (502-859); follow up > 5 yrs | Whole Blood, PBMsC, CD4+ and CD8+ T cells | Hocini et al 2019 – (45) |
| Microarray | HIV progressors in absence of ART(n=11), VC (n=9), EC (n=12) | VC: VL < 5000; EC: VL <50 | CD4+ T cells | Morou et al, 2019 – (57) |
| RNA-seq | EC (n=58), VC (n=73), HIV negative (n=35) | EC: VL < 50; VC: VL < 2000 - > 50; both follow up > 12 months | anti-CD3/CD28 activated CD4+ | Gonzalo-Gil et al, 2019 – (30) |
| RNA-seq | HIV progressors in absence of ART (n=7), HIV progressors on ART (n=7), vLTNP (n=8), EC-LTNP (n=8) | LTNP: follow up 10 yrs; CD4+ > 500; vLTNP: VL < 10 000; EC-LTNP: VL und. or <2000 in less 25% of determinations | PBMCs | Diez Fuertes et al, 2019 – (44) |
| RNA-seq | EC (n=33), HIV progressors in absence of ART (n=15) | EC: follow up > 1 yr; VL < 75 (one blip allowed always < 1000) in > 3 consecutive determinations. Also HIV+ > 10 yrs with >90% VL < 400. | PBMCs | Paim et al, 2019 – (47) |
| Integrative Meta- analysis: Microarray | LTNP (n=58), HIV progressors on ART (n=54), HIV progressors in the absence of ART (n=105), HIV negative (n=22). | Those used in Hyrcza, 2007; Rotger, 2010; Rotger, 2011; and Vigneault, 2011. | CD4+ T cells | Lee et al, 2019 – (62) |
| Integrative Meta- analysis: Microarray | viremic non progressors (VNPs) (n=5), EC (n=34), and HIV progressors (n=35). | Those used in Hyrcza, 2007; Quigley, 2010; and Klatt, 2014. | Whole blood, CD4+ and CD8+ T cells | Ding et al, 2019 – (79) |
| Microarray | HIV progressors in the absence of ART(10), HIV progressors on ART (10), EC(10), HIV negative (10) | EC: stable CD4+ T cell counts; VL < 50 in 3 consecutive measurements, > 1yr | Resting memory CD4 T cells | Garcia et al, 2020 – (60) |
| RNA-Seq | EC(n=2), HIV progressors (n=2), HIV negative (n=2) | EC: VL = Undetectable | CD4+ T cells | Chen, Wu, Lu, 2020 – (66) |
| RNA-seq | HICs: Neutralizers (n = 46) and non-neutralizers (n=15)(with or without bNAbs against HIV-1 in plasma, respectively) | HICs: CD4+ 1 685 - 385; VL < 2000; median of follow-up 5 years | mDCs, CD4+, B cell, Monocytes | Martín-Gayo et al, 2020 – (58) |
| RNA-seq | EC-LTNP (n=8), vLTNP (n=8), HIV progressors in absence of ART (n=7), HIV progressors on ART (n=7). | LTNP: follow up 10 yrs; CD4+ > 500; vLTNP: VL < 10 000; EC-LTNP: VL und. or <2000 in less 25% of determinations | PBMCs | Ayala-Suarez et al, 2020 – (54) |

CD4+ count: cells/µL or cells/mm3; viral load (VL): copies HIV RNA/mL; VL und.: viral load undetectable (<75 or <50). All LTNP/HIC individuals did not receive ART

**Supplementary Table 2. Differentially expressed genes and pathways of relevance found in transcriptomic studies listed by reference.**

| **Reference** | **Cell type** | **Compared groups (compared vs basal)** | **DEGS** | **Pathways** |
| --- | --- | --- | --- | --- |
| Hyrzca et al, 2007 – (63) | CD4+ T cells, CD8+ T cells | (LTNP + HIV-) vs (Acute infection + chronicly infected) | **Up**: ISGs (IFI44L, DNAPTP6, HERC6, IFI44, OAS family, among others). | Interferon response, DNA replication, cell cycle regulation, and apoptosis. |
| Quigley et al, 2010 – (71) | CD8+ T cells | HIC vs untreated progressors | **Up**: RASA1 **Down**: BATF, STAT1, IRF9, IFI44. | Interferon response, MHC expression, mRNA transcription, protein translation. |
| Salgado et al, 2011 – (55) | CD3+ T cells | LTNP vs typical progressors | **Up**: PDPK1, TLE4, SHOC2, IL17RA, IL1RAP, BMPR2, ROCK1, RDX, PDGFB, KLHL5. | Cytokine-cytokine receptor interaction, regulation of actin cytoskeleton, focal adhesion processes, negative regulation of apoptosis. |
| Rotger et al, 2011 – (5) | CD4+ T cells, CD8+ T cells | Rapid Progressors vs EC | **Up**: ISGs (IFI44, IFI44L, MX1, EIF2AK2, IFI6, LY6E, TRIM22), SOCS1. | Interferon Response. |
| Vigneault et al, 2011 – (68) | CD4+ T cells | EC vs HIV- | **Up**: TRAF4, SMAD7, CDKN1A, MAP3K8. | Activation of NF-kB and JNK, cell cycle progression and cell proliferation, Th cells differentiation, IFNG and TNFA expression. |
| Wu, 2011 – (56) | Whole Blood, PBMCs, CD4+ and CD8+ T cells | LTNP vs progressors under ART | **Up**: MEK3, NFKBIA, JNK2, MKK7, PIK3R1, PIK3CA, PPP2CA. **Down**: ATP6V1D, ACTA2, PSMB2, PSMA5. | MAPK p38 pathway, NFKB activation, JNK/MAPK pathway, granule cell survival pathways, AKT and WNT signaling pathways. |
| Swaminathan et al., 2012 – (69) | CD4+ T cells | LTNP vs HIV- | **Down**: let-7 miRNA family members | - |
| Witwer, 2012 – (52) | PBMCs | EC vs untreated progressors | **Up**: miR-31-5p, miR-29a, miR-150. **Down**: miR-155. | T cell differentiation and activation, latency induction and viral replication control. |
| Wu et al, 2013 – (76) | Monocytes | LTNP vs progressors under ART | **Down**: IL1B, IL8. | Cell signaling, cell cycle and apoptosis, cytoskeleton and cell migration, phagocytosis and metabolism. |
| Klatt et al, 2014 – (46) | Whole Blood | VNP vs typical progressors | **Up**: IRF7, OAS2, GBP2, IRF1, STAT2, HSPD1, ITGB3, KLRAP1. **Down**: MX2, IFIT5, IFI44, DDX58, IFI27, OASL, MX1, ISG15, CD9, IFNAR1, STAT5B, CXCL3. | Leukocyte migration, leukocyte activation, immune effector process, activation of immune response, antigen processing and presentation. |
| De Masson et al, 2014 – (65) | CD4+ (subpopulations) | EC-LTNP vs HIV- | **Up**: AHNAK, PIK3R1, IFITM1, PYHIN1, PRDM1. **Down**: FOXO3, PDE4D, FTSJD2, IFI6, DDX21. | TCR activation and costimulatory signaling, Type I IFN-signaling pathways. |
| Xu et al., 2014 – (75) | PBMCs | Non-progressors vs progressors | **Up**: ISGs, SOCS1, APOBEC3G, LY6E. | - |
| Luque et al., 2014 – (48) | PBMCs | LTNP vs HIV- (excluding DEGs from progressors under ART vs HIV-) | **Up**: CAMP, CSTG, CXCL10, HSH2D, STAT5B. **Down**: FCER1A, PPBP, ITGB2, SPON2, ILK. | Survival, inhibition of apoptosis, T-cell activation, proliferation, Immune cell trafficking. |
| Reynoso et al, 2014 – (51) | Plasma | EC vs untreated progressors | **Up**: mir-29b-3p, miR-33a-5p, miR146a-5p. | Apoptosis, viral DNA integration, viral entry. |
| Egaña-Gorroño et al, 2014 – (53) | PBMCs | (EC + HIV-) vs (untreated progressors + progressors under ART) | **Up**: miR-29b-3p, miR27b, miR-221. **Down**: miR-146a, miR-155. | Apoptosis, viral transcription, immune activation and inflammation. |
| Egaña-Gorroño et al, 2016 – (74) | CD8+ T cells | EC vs HIV- / VC vs HIV- / untreated progressors vs HIV- | **Up**: miR-4505. | CD8 T cell activity. |
| Liao et al, 2017 – (61) | CD4+ T cells | LTNP vs typical progressors | **Up**: CCL22, LILRB3, CCL7/MCP-3, TRAP1, TUBB1,KLRG1. **Down**: TMPO, BST2, RBX1, CCNA2, OAS2, FOXM1, EZH2, PAFF1. | Immune system process, response to virus stimulus, and inflammatory response, RIG-I-like receptor signaling pathway. |
| Zhang et al, 2017 – (78) | Whole Blood, CD4+ and CD8+ T cells | LTNP | **Up**: CCR7, NELL2, MMD, SORL1, FAM46C, RBM38. **Down**: ISG15, LY6E, MX1, OAS1, OAS2, OAS3, OTOF, RNASE2, USP18, XAF1. | Cytokine-cytokine receptor interaction, MAPK signaling pathway. |
| Chowdhury et al., 2018 – (70) | CD8+ T cells | EC vs progressors under ART | **Up**: IL-2, IL-7, IL-15, TNF-α , IL-1β. | eIF2, mTOR and PI3K signalling. |
| Buggert et al., 2018 – (72) | CD8+ T cells | Lymph nodes T CD8 cells vs peripheral blood T CD8 cells from EC. | **Up**: TUBAP2, ANKLE2, CD3D, FTH1 **Down**: CFL1P2, CFL1, HLA-E, HLA-DBP1. | IFN response, cell location, signal transduction. |
|  |  | CD69+ vs CD69- CD8 T cells from EC | **Up**: KLDR1, CD300A, ZEB2, BCL2. **Down**: S1PR1, FOXP1, CCR7, CXCR4. | Cytolisis, cell killing, cell signalling. |
| Martín-Gayo et al, 2018 – (77) | mDCs | Different mDC cluster from 3 EC after HIV infection | **Up**: IFIT3, CXCL10, CD274, FCGR1A, STAT1, IFITM3, IFI6. | Innate recognition, DC maturation, interferon and TLR signaling, EIF2 signalling, Th1 pathway |
| Zhang et al., 2018 – (49) | PBMCs | EC ♂ vs EC ♀ | **Down**: JUN, FOS, FOSB, SOCS1. | Immune activation. |
|  |  | EC vs untreated progressors | **Up**: CCL4, CCL7, MIP1B, CD40, CD40L. **Down**: FASL, TRAIL, PD1, SIGLEC1, CXCR6. | Cytokine signaling, correceptor signaling, apoptosis. |
| Nguyen et al., 2019 – (73) | CD8+ T cells | EC vs untreated chronic progressors (HIV-specific CD8 T cells from lymph nodes) | **Up**: CCL5, TNF, RNASE1, IL32. **Down**: PRF1, GZMB. | Immune response, immune system process, defense response. |
| Hocini et al 2019 – (45) | Whole Blood | HIC vs progressors under ART | **Down**: TLR8, TREM1, IL-8/CXCL8, CXCR1, CXCR2, FCGR3A/FCGR3B, FCGR2A. | Innate immunity and NK cell signaling, T cell activation and inflammation. |
|  | PBMCs | HIC vs progressors under ART | **Down**: HBA1/HBA2, HBB, HBG1, HBG2, IL1B, IL-6, CXCL5, CXCL1, CD14. | Inflammation, granulocyte adhesion, diapedesis. |
|  | CD8+ T cells | strong responders HICs vs weak responders HICs | **Up**: CXCR1. **Down**: CXCL8, IL1B, IRAK, TYROBP, FCER1G. | T cell response, cytotoxicity of leukocytes, killing NK cells, activation of leukocytes. |
| Morou et al, 2019 – (57) | CD4+ T cells | EC vs chronic progressors (Gag-reactive cells) | **Up**: BCL6, RORC, TBX21, IL17F, IL22. | Th1 differentiation, Th17 differentiation, cell proliferation. |
| Gonzalo-Gil et al, 2019 – (30) | anti-CD3/CD28 activated CD4+ | HICs vs HIV- | **Up**: MX1, OAS1, OAS3, IFI44, IFI44L. **Down**: CCR2, CCR5. | Interferon response, cytokine signaling. |
| Diez Fuertes et al, 2019 – (44) | PBMCs | EC-LTNP vs progressors under ART | **Up**: eEF1 complex, XRCC6. **Down**: RP11.288L9. | Intracelular calcium mobilisation, repression of viral transcription. |
|  |  | EC-LTNP vs vLTNP | **Up**: CDKN1A, TNF, IER3, GADD45B. | Modulation of HIV-1 transcription. |
|  |  | LTNP vs typical progressors | **Up**: ANKRD54. **Down**: VWA8, IGHA2. | - |
| Paim et al, 2019 – (47) | PBMCs | EC vs untreated progressors | **Down**: TRAILshort, PLSCR1, GIMAP4, NMI, TLR7. | Apoptosis, IFN response. |
| Lee et al, 2019 – (62) | CD4+ T cells | LTNP vs untreated progressors | **Up**: PHLDA1. | Cell adhesion. |
|  |  | LTNP vs progressors under ART | **Down**: ACTB, ACTG1. | Cytoskelleton. |
|  |  | LTNP vs HIV- | **Up**: THSB1, RBM38. | MAPK signaling pathway, cell adhesion, stabilization of mRNA. |
| Ding et al, 2019 – (79) | Whole blood, CD4+ and CD8+ T cells | Non-progressors vs progressors | **Up**: CMPK1, CBX7, EIF3L, EIF4A, ZNF395, CD9, METTL9. **Down**: CD38, LAG3, ISGs. | Type 1 interferon signaling pathway, interferon-gamma mediated-signaling pathway innate response, ribosome components and translation. |
| Garcia et al, 2020 – (60) | Resting memory CD4 T cells | EC vs untreated progressors | **Down**: IFIT1, IFIT3, LGALS9. | Immune response, NKT cell differentiation. |
|  |  | EC vs progressors under ART | **Up**: KLF12, ABCB1, SLC4A10. | Lymphocite anergy, humoral immune response, mucosal immune response, T cell activation/proliferation/functionality, NK or NKT cells differentiation/citotoxicity. |
| Chen, Wu, Lu, 2020 –(66) | CD4+ T cells | EC vs untreated progressors | **Down**: HDAC6, ATM, MAPK8, IRF3. | Negative regulation of IFNb production, regulation of defense response to virus, negative regulation of type I interferon production, RIG-I like receptor signaling pathway, antigen processing and presentation, NF-kB signaling pathway. |
| Martín-Gayo et al, 2020 – (58) | mDCs | HICs with broad neutralizing antibodies vs non-neutralizers. | **Up**: CD40LG, CD24, E2F1, KDM5A, TREM1. | BCR Signalling, CD28 Signalling in T Helper cells, CD40 Signalling, NFKB Signalling. |
|  | CD4+ T cells |  | **Up**: MAPK4K4, MAPK1. | Death receptor, Lymphotoxin Beta R and BCR Signalling, Induction of apoptosis by HIV. |
| Ayala-Suarez et al, 2020 – (54) | PBMCs | LTNP vs typical progressors | **Up**: miR-99a-5p. **Down**: miR-451a, miR18a-5p. | Phagosome, leukocyte transendothelial migration, antigen processing, ubiquitination and proteasome degradation, Vpu mediated degradation of CD4. |
|  |  | EC-LTNP vs vLTNP | **Up**: miR-146a-5p, miR155-5p. **Down**: miR-29b-3p. | NF-kB signaling pathway, TLR4 signaling pathway, viral translation. |

LTNP: Long-term non-progressors. HIV-: HIV negative patients. ISG: Interferon Stimulated Genes. HIC: HIV controllers. EC: Elite controllers. ART: Antiretroviral therapy. VNP: Viremic non-progressors. DEGs: Differentially expressed genes. VC: Viremic controllers. mDC: myeloid dendritic cells.
